# Epidemiology of Endometriosis in Spain and Its Autonomous Communities: A Large, Nationwide Study

**DOI:** 10.3390/ijerph18157861

**Published:** 2021-07-25

**Authors:** Almudena Ávalos Marfil, Enriqueta Barranco Castillo, Raúl Martos García, Nicolás Mendoza Ladrón de Guevara, Maryna Mazheika

**Affiliations:** 1 Department of Obstetrics and Gynecology, University of Granada, 18016 Granada, Spain; almuavalosmarfil@gmail.com (A.Á.M.); ebc@ugr.es (E.B.C.); nicomendoza@ugr.es (N.M.L.d.G.); 2 Gynaecology and Obstetrics Unit, “San Cecilio” University Hospital, 18012 Granada, Spain; 3 Red Cross Nursing School, University of Seville, 41009 Seville, Spain; rmartosg@hotmail.com

**Keywords:** endometriosis, epidemiology, diagnosis

## Abstract

A retrospective population-based study aimed to assess the incidence of endometriosis in the general population in Spain and in each of its autonomous communities. The authors used the incidence of diagnosed endometriosis in the minimum basic dataset at discharge in the national hospital discharge registry of Spain. This analysis was carried out with hospital data with a diagnosis of endometriosis (International Classification of Diseases (ICD)-9 code 617.xx and ICD-10 code N80.xx) and covered the period from 1 January 2014 to 31 December 2017 and a population of 12,775,911 women of reproductive age (15–54 years). The data were then analyzed at the national level and separately for each autonomous community. This nationwide Spanish study estimated the overall incidence of endometriosis among autonomous communities in Spain to be 16.1 per 10,000 women (range, 6.8 to 24). The mean age of the 20,547 women diagnosed with endometriosis during the study period was 36.8 ± 5.4 years. The types (proportions) of endometriosis were uterine (28.4%), tubo-ovarian (35.2%), peritoneal (8.1%), vesical (6.8%) and intestinal (3.2%) endometriosis. Further studies are needed to assess the reasons for the decrease in the observed incidence and for the significant differences in the regional incidence rates of this disease.

## 1. Introduction

Endometriosis is a painful disorder characterized by the presence of endometrial glands and stroma outside of the uterine cavity [1]. The main symptoms are pelvic pain and infertility [1,2]; however, the symptoms of endometriosis vary in intensity and are not always proportional to the extent of disease, as endometriosis can be asymptomatic in many cases [3]. The main types of endometriosis are ovarian endometriosis, uterine endometriosis, peritoneal endometriosis and vesical endometriosis [3].

Estimates of the incidence of endometriosis suggest that it could affect up to 10% [2,4] of women of reproductive age worldwide and approximately 30–50% of women with symptoms suggestive of endometriosis [5,6,7], but the limitations of these estimates are that they are usually determined based on data from patients. Diagnosis is difficult and is mainly based on the symptoms, vaginal ultrasound and MRI imaging, and is delayed for an average of 10 years between the first symptom and diagnosis [3,5]. The true incidence of endometriosis in a global and community setting has not yet been determined [6], and information from epidemiological studies on endometriosis is limited.

The objective of this study was to assess the incidence of diagnosed endometriosis in the general population in Spain and in 19 Spanish autonomous communities. Currently, there is a gap in global data on endometriosis, particularly in Spain, as well as a limited amount of data obtained in population studies around the world.

## 2. Materials and Methods

### 2.1. Study Setting

The hospital discharge registry, also known as the CMBD (minimum basic dataset at discharge), is the largest administrative database for hospitalized patient data in Spain [8]. Its basic structure was approved in 1987 by the Interterritorial Council, following the recommendations made by the Council of Europe. Currently, it is required that hospital data, patient baseline data and clinical data from admissions from both the private and public sectors is entered into this database for inclusion in the national statistical plan. Proposed indicators can be analyzed based on the information contained in the CMBD, without having to access additional information sources, thus ensuring that we could estimate national endometriosis incidence rates with this single database. The hospitals in the autonomous communities are submitting this information; therefore, we used the Registry of Activity of Specialized Care, which is a dataset within the CMBD. All diagnoses made during each admission are coded according to the 9th and 10th editions of the International Classification of Diseases (ICD-9 and ICD-10). Population data are collected from the National Institute of Statistics: study population, data analysis and ethical approval [9].

### 2.2. Study Population and Ethical Approval

This is a retrospective population-based study of patients discharged from all hospitals in Spain between 1 January 2014 and 31 December 2017. Women aged 15–54 years, diagnosed with endometriosis new cases only, were identified according to ICD diagnostic codes (until 2014, ICD-9 code 617.xx and apart from 2016, ICD-10 code N80.xx, codes do not overlap), and their data were extracted from the CMBD. Initially, we started with the data CMBD, and categorized these data by year and the aforementioned autonomous communities (in addition to the autonomous cities of Ceuta and Melilla). These data included the absolute number of patients diagnosed with endometriosis and the endometriosis type. The 20,547 women with any form of endometriosis were included who met the case ascertainment criteria for prevalent endometriosis. Concerning the validity of case ascertainment, all women in this population had a diagnosis from a gynecologist or surgeon, data were sent and the hospital assigned a code according to the ICD diagnostic codes, as we have pointed out. To generate a map of the incidence of endometriosis in each autonomous community, the patient was grouped according to the autonomous community in which they lived. Only the first hospitalization was considered, and rehospitalizations were studied separately. Clinical trial guidelines (STROBE) were used in the design of this study. This study was approved by the Committee of Ethics of the University of Granada. Written informed consent was not required, as this was a retrospective database analysis.

### 2.3. Study Variables

Sociodemographic data, namely the woman’s age and autonomous community of residence, were obtained from the CMBD. For all patients with a diagnosis of endometriosis, we identified the type of endometriosis by means of the ICD diagnostic code (uterine endometriosis (617.0; N80.0), tubo-ovarian endometriosis (617.1; N80.1 y 617.2; N80.2), peritoneal endometriosis (617.3; N80.3), vesical endometriosis (617.8; N80.8), intestinal endometriosis (617.5; N80.5) and endometriosis, site unspecified (617.9; N80.9).

### 2.4. Statistical Methods

Data obtained from the national database were transferred to SPSS 22 (IBM, Armonk, NY, USA) [10] for subsequent statistical analysis, and all statistical analyses were performed using this software. The variable indicating which of the 19 autonomous communities each woman lived in was categorized to evaluate the incidence for each autonomous community by study year and for the entire study period (2014–2017).

In the initial descriptive analysis of the study population, quantitative variables are presented as the mean (m), standard deviation (SD), and 95% confidence interval (95% CI); qualitative or categorical variables are presented as a percentage (%).

The annual incidence in each autonomous community was calculated based on the diagnosed cases and the existing population in each year. The period incidence (the frequency of a disease in a period of time) was calculated by adding the cases in the period between 2014–2017 and multiplying this number by 10,000.

In the statistical analysis of the relationships between different variables, *p* < 0.05 indicated a statistically significant or relevant relationship. For quantitative variables, the Pearson correlation coefficient was determined, and for qualitative variables, the chi-square test was used. To examine the relationships between qualitative variables (autonomous communities) and quantitative variables (incidence), nonparametric tests (the Kruskal–Wallis H test for more than 2 categories) were used after performing the Kolmogorov–Smirnov test to examine the normality of data.

Finally, the relationship between years of study of the annual incidence data of diagnosed cases of endometriosis as a whole and in each autonomous community, in addition to in the cities of Ceuta and Melilla, was analyzed by means of a linear model for repeated measures.

## 3. Results

In Spain, the total number of women of reproductive age (15–54 years) from 1 January 2014 to 31 December 2017 was 12,775,911. Among them, 20,547 patients were diagnosed with endometriosis, and the mean age of these patients was 36.8 ± 5.4 years.

**Figure 1:**
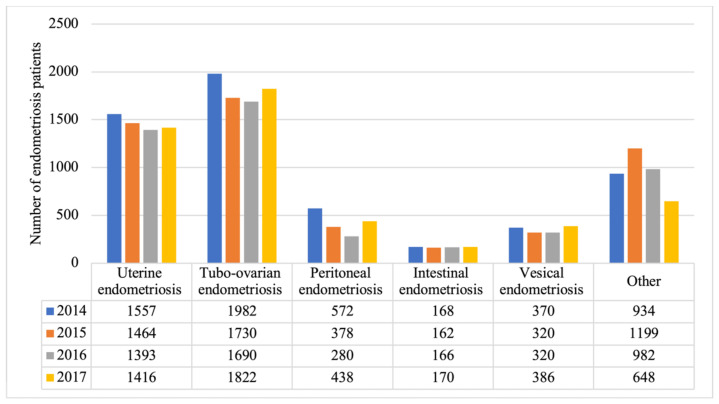
Number of endometriosis patients according to type of endometriosis (2014–2017).

Incidence rates were also estimated according to the following types of endometriosis in each year of the study (Figure 1), and average incidence rates for the study period (2014–2017) were as follows: uterine endometriosis, 28.4%; tubo-ovarian endometriosis, 35.2%; peritoneal endometriosis, 8.1%; vesical endometriosis, 6.8%; intestinal endometriosis, 3.2%; and of other type, 18.31%.

**Figure 2:**
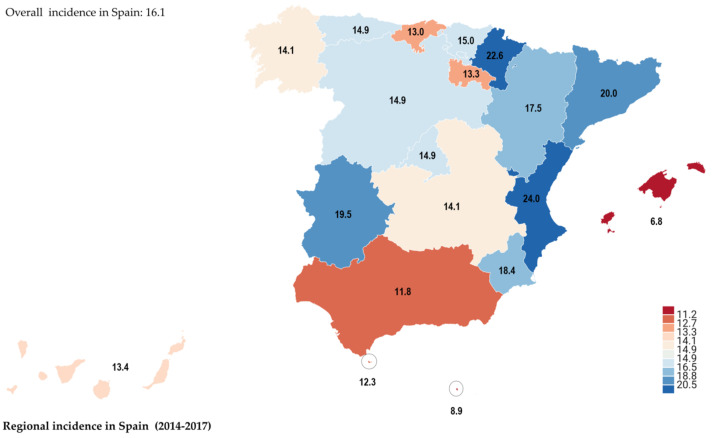
Incidence (cases per 10,000 women) from 2014–2017.

Approximately 5000 women in Spain were diagnosed with endometriosis during each year of the study. The overall incidence of endometriosis in Spain was 16.1 cases per 10,000 women, and the rates ranged from 6.8 to 24 per 10,000 women among the autonomous communities, with the incidence of endometriosis being higher in Valencian (24), Navarre (22.6), Catalonia (20), Extremadura (19.5) and Murcia (18.4) (Figure 2). The annual incidence of endometriosis in Spain from 2014 to 2017 decreased from 4.4 to 3.9 per 10,000 women.

To this end, a significant difference was observed in the data obtained for each autonomous community over the study period (*p* = 0.000) and for the national average over the same period (*p* = 0.000). These data were corroborated with the calculation of the annual percent variation in the same data, where the national average percent variation between 2014–2017 ranged between 137.5% and −65.5%.

## 4. Discussion

### 4.1. Main Findings

Published database studies worldwide have indicated that endometriosis is very difficult to diagnose and that diagnosis is frequently delayed [3,6,11,12,13]. Moreover, some studies have shown that asymptomatic forms of endometriosis exist [1,3,11]. Therefore, the assessed incidence of endometriosis in the general population may be an underestimate because of these factors. The incidence calculated from discharge data is notable because it concerns only women who were hospitalized and diagnosed with endometriosis. Because conditions such as chronic pelvic pain, interstitial cystitis, fibromyalgia and irritable bowel syndrome may cause symptoms that are similar to those of endometriosis, the diagnosis of endometriosis may be delayed due to misdiagnosis [11,14,15,16,17].

The results of this national population-based study indicate that the incidence of endometriosis in the general population of Spain is 16.1 per 10,000 women. The available published data from multiple countries around the world, comprising incidence estimates of diagnosed disease, show wide variations in incidence, ranging from 0.2% to 71.4% depending on the population examined (e.g., patients with menorrhagia or abnormal uterine bleeding; dysmenorrhea or pelvic pain; dyspareunia; or infertility) [4,14,15].

Formal estimates of the incidence of pelvic endometriosis in the female population in Spain are lacking [6]. In the literature, there are some studies in other countries that are somewhat similar in terms of study design to our study [18,19,20,21]. In Israel, the estimated crude point incidence of endometriosis was 10.8 per 1000 women aged 15–55 years [18]. A French nationwide study that estimated the incidence of hospitalizations for a main or associated diagnosis of endometriosis in each region of the country reported frequencies ranging from 0.4% to 1.6% among women aged 15–49 years [19]. Our study revealed some marked differences among the autonomous communities with regard to the incidence of endometriosis in the general population. In our study, the mean age at diagnosis of the patients was 36.8 ± 5.4 years. The highest incidence (12.8 per 1000 women) was observed in a German population of women aged 35–44 years [22], and the average age at diagnosis was 35.1 years in the UK [11].

Notably, the annual incidence of endometriosis in Spain from 2014 to 2017 decreased from 4.4 to 3.9. It seems unlikely that this trend is due to improvements in imaging techniques because only the most symptomatic patients are hospitalized and eventually diagnosed, while asymptomatic women usually are not [3]. With a type such as uterine adenomyosis, it is recognized that some women are asymptomatic [23,24]. On the other hand, the diagnostic process usually takes 5–10 years, and the lack of formal control of pain relief after diagnosis and treatment means that women are actively seeking information from other sources [25]. It should also be noted that symptoms, when present, can be severe and affect many organ systems, including the gastrointestinal and urinary systems and/or need to be addressed concurrently for optimal patient care [26]. Tolerance to symptoms such as pain or infertility is decreasing in society as quality of life is improving [12]. Our study was not able to address these matters.

In addition to the annual incidence rates for endometriosis overall, we determined the relative frequencies of the different types of endometriosis resulting in hospitalizations by means of an unselected population or, more specifically, among the entire population of Spain. The most frequent types were tubo-ovarian and uterine endometriosis. In the literature, there are only a few studies based on nationwide data that describe the proportion of cases related to each pelvic organ involved. After performing comparisons of our findings with those from other countries to see if the relative proportions and types of endometriosis were the same, we found that the different endometriosis types may be related to different etiologic factors in different geographic areas. In France, the most frequent types were ovarian and peritoneal endometriosis [19]. In a Canadian population, uterine endometriosis accounted for 28.29% of cases, ovarian endometriosis accounted for 27.44% of cases, and other endometriosis types accounted for 44.27% of the cases diagnosed among 47,021 women admitted to the hospital due to endometriosis over 5 years [21]. In a United States population-based study from 2006–2015, the overall adenomyosis incidence was 1.03%, or 28.9 per 10,000 woman-years [27]. The estimated incidence of adenomyosis in northeast Italy represented 28% of all diagnoses [20].

In a UK hospital-based study, the incidence of diagnosed endometriosis in the general population was 1.5%, which demonstrated that the symptoms associated with endometriosis are relatively uncommon in women without endometriosis and are associated with endometriosis-specific symptoms, such as abdominopelvic pain, dysmenorrhea, menorrhagia, and ovarian cysts [11]. Further characterization of this cohort may help to understand the incidence of endometriosis in symptomatic versus asymptomatic patients and to promote earlier diagnosis in clinical practice.

The financial burden of endometriosis on the healthcare system is substantial, with average annual costs of endometriosis estimated at US $12,419/woman affected (€8559–10,599) [28]. In a recent study, the hospital cost associated with endometriosis was approximately $30 million Canadian dollars (US $29.56 million) per year [21]. These studies have generally classified healthcare cost into direct and indirect costs. Direct costs typically include inpatient, outpatient, surgery, drugs, medical services and other healthcare services, whereas indirect costs may include loss of productivity, absenteeism, short- and long-term disability, loss of leisure time and adverse effects on quality of life.

### 4.2. Strengths and Limitations

The key strength of our study was that it was a retrospective population-based study with comprehensive longitudinal data from the whole population of women with endometriosis in Spain. Variations in environmental factors, genetic profiles and access to healthcare services should be considered when comparing these results with those from other countries [1,29].

There were also some limitations to our study. Given the reliance on ICD-9 and ICD-10 diagnostic codes for the selection of women with endometriosis, there was potential for under-detection-related biases in the estimates of endometriosis incidence even though this disease, when diagnosed, is considered serious by gynecologists and thus routinely coded. Nonetheless, coding quality is checked by medical information professionals in each hospital to correct diagnoses and may still vary among institutions. Furthermore, as many patients are not admitted to the hospital, this study assessed only the incidence of hospital registrations for endometriosis, not the incidence of endometriosis in the whole population.

Another limitation was the difficulty in analyzing all of the comorbid factors that may explain differences among the autonomous communities. Further research may be needed, including local investigations to collect information that was not available in our dataset.

### 4.3. Interpretation

Estimates of the frequency of endometriosis vary widely. The incidence in Spain was more or less the same as that in clinical studies. There may be some reasons for variations in incidence among the autonomous communities. As discussed above, the global incidence of the condition is approximately 10% [4,6,18,19]. Although there is no consistent information on the disease incidence, temporal trends suggest that it is higher among women of reproductive age [1,19,20,21,30,31]. This can be partly explained by changing reproductive habits (increased maternal age and increased use of artificial reproductive technology) [2]. Factors and symptoms of endometriosis should be taken into account to understand the trend toward an increased incidence of endometriosis [3].

## 5. Conclusions

This is the first detailed epidemiological study of endometriosis in Spain and the first nationwide study to estimate the incidence of endometriosis in its autonomous communities. The results show that the incidence of endometriosis in Spain is 16.1 per 10,000 (ranging from 6.8 to 24 among the autonomous communities). This study also provides information on the relative proportions of the different types of endometriosis. Further studies are needed to assess the reasons for the increasing incidence of endometriosis and for the significant differences in the regional incidence of this disease. Our research team intends to conduct a follow-up study to estimate the cost of endometriosis in Spain. Nevertheless, the true incidence of endometriosis in real-world settings has not been clearly established.

## Data Availability

The data presented in this study are available on request from the corresponding author. The data are not publicly available due to due to a request from the Registry of Activity of Specialized Care, which is a dataset within the CMBD (minimum basic dataset at discharge). The population data available in a publicly accessible repository. The data presented in this study are openly available in [9].
